# Biomarkers in Bladder Cancer Surveillance

**DOI:** 10.3389/fsurg.2021.735868

**Published:** 2021-09-28

**Authors:** Sukumar S. Sugeeta, Anand Sharma, Kenrick Ng, Arvind Nayak, Nikhil Vasdev

**Affiliations:** ^1^Department of Medical Oncology, Mount Vernon Cancer Centre, Northwood, United Kingdom; ^2^Department of Urology and Surgery, Lister Hospital, East and North Herts NHS Trust, Stevenage, United Kingdom; ^3^School of Life and Medical Sciences, University of Hertfordshire, Hatfield, United Kingdom

**Keywords:** biomarker, bladder cancer, surveillance, non-muscular invasive bladder cancer, cancer screening

## Abstract

**Aim:** This is a narrative review with an aim to summarise and describe urinary biomarkers in the surveillance of non-muscle-invasive bladder cancer (NMIBC). It provides a summary of FDA-approved protein biomarkers along with emerging ones which utilise genetic, epigenetic and exosomal markers. We discuss the current limitations of the available assays.

**Background:** Current guidelines advice a combination of cystoscopy, imaging,and urine cytology in diagnosis and surveillance. Although cytology has a high specificity, it is limited by low sensitivity particularly in low grade tumours. There are six FDA-approved urinary assays for diagnosis and surveillance of bladder cancer. They have shown to improve sensitivity and specificity to be used alongside cytology and cystoscopy but have a lower specificity in comparison to cytology and false positives often occur in benign conditions. Recent developments in laboratory techniques has allowed for use of markers which are RNA-, DNA-based as well as extracellular vesicles in the past decade.

**Methods:** Using the PubMed/Medline search engines as well as Google Scholar, we performed an online search using the terms “bladder cancer,” “non-muscle invasive bladder cancer,” and “urine biomarkers” with filter for articles in English published up to May 2021. Systematic reviews and original data of clinical trials or observational studies which contributed to the development of the biomarkers were collated.

**Results:** Biomarkers identified were divided into FDA-approved molecular biomarkers, protein biomarkers and gene-related biomarker with a table summarising the findings of each marker with the most relevant studies. The studies conducted were mainly retrospective. Due to the early stages of development, only a few prospective studies have been done for more recently developed biomarkers and limited meta-analyses are available.Therefore a detailed evaluation of these markers are still required to decide on their clinical use.

**Conclusion:** Advancements of analytical methods in BC has driven the research towards non-invasive liquid-based biomarkers in adjunct to urine cytology. Further large prospective studies are required to determine its feasibility in a clinical setting as they are not effective when used in isolation as they have their limitation. With the ongoing pandemic, other than reduction in costs and increased accuracy, the need for biomarkers to cope with delay in cystoscopies in diagnosis and surveillance is crucial. Thus clinical trials with direct comparison is required to improve patient care.

## Introduction

Bladder cancer accounts for 90–95% of urothelial cancers. It is the eight most common cancer in women and fourth most common cancer in men (1). Most cases present with non-muscle invasive bladder cancer (NMIBC) and at early stages this carries a favourable prognosis. However, NMIBC accounts for 75% of cases and has a high recurrence rate of 80% in high-risk lesions and up to 50% in low risk. The 5 year survival rate is 94% if detected early and therefore early detection is imperative as intervention drastically influences overall survival (2).

Currently, bladder cystoscopy in combination with imaging of the upper urinary tract along with voided urine cytology as part of surveillance. NICE guidelines recommend cystoscopy to be done every 3 months for the first 2 years then every 6 months for the next 2 years then once a year thereafter. Cystoscopy is associated with complications such as a urinary tract infection, haematuria, pain. The utilisation of both imaging and cystoscopy is not effective in detecting smaller lesions. Urine cytology remains the most widely used non-invasive method for both diagnosis and surveillance of BC. Studies have shown a high specificity of 86% but this is limited by its low sensitivity of 48% as there is subjective nature when grading urothelial carcinoma on urine samples resulting in poor inter-observer variability (3). Although routinely used as the standard of truth for assessment of diagnostic accuracy, it is well-recognised that traditional cystoscopy with use of white light can lead to missing lesions that are present but not visible. New technologies exist to improve tumour visualisation. A recent study compared the use of blue light flexible cystoscopy with hexaminolevulinate (HAL, Hexvix®, Photocure ASA) with white light flexible cystoscopy for the detection of bladder cancer during surveillance, finding that 20.6 % (95% CI 11.5–32.7, *p* < 0.0001) of patients with recurrent cancer was seen only with blue light (4). The fact that a significant proportion of recurrences are missed under white light cystoscopy should be taken into consideration when assessing the sensitivity of new markers.

Urinary biomarkers play an important role in the future of precision medicine given the limitations of the current modalities being used given the specificity and sensitivity and need for invasive procedures to allow for surveillance. There is also a significant impact due to costs involved to healthcare services given the frequency and reliance on cystoscopy at present. This has led to the development of several non-invasive biomarkers which are now FDA approved. This is now particularly relevant with regard to low and intermediate risk patients who have had cystoscopies deferred with the ongoing pandemic. UroFollow is a multi-centre prospective trial exploring follow up using urine biomarkers in comparison to standard of care to explore if non-invasive methods are sufficient or patients with low grade or pTa G1–G2 BC (5). In addition to diagnostic accuracy, biomarkers need to be reproducible tests, affordable and easily implementable. This review's aim is to summarise biomarkers which have been identified for use in BC surveillance which are FDA-approved, commercially available and potential biomarkers in development.

## FDA-Approved Molecular Biomarkers

The United States Food and Drug Administration (FDA) have currently approved 6 urinary assays to use alongside cystoscopy for diagnosis and surveillance. These include BTA stat (Polymedco), BTA TRAK (Polymedco), NMP22 enzyme linked immune-sorbent assay (ELISA) (Matritech), NMP22 BladderChek Test (Alere), uCyt (Scimedx) and UroVysion (Abbott Molecular).

### NMP22

Nuclear matrix proteins are non chromatin structures which play several roles from DNA replication to gene expression and contributes to the infrastructure of the cell nucleus. During replication in healthy cells, NMP22 regulates the distribution of chromatin to daughter cells and this is normally at low levels. In urothelial tumours, levels of NMPs are high due to cell turnover from tumour apoptosis. NMP22 is one of them and it is the most investigated as an assay in both diagnosis and recurrence of bladder cancer.

Two modes of detection were FDA approved for both diagnosis and surveillance. NMP22 were initially detected with quantitative ELISA in a laboratory where cut-off values were utilised. The second was a qualitative point-of-care test, the NMP22 BladderChek where monoclonal antibodies are used to detect raised NMP22 levels in BC.

In 2015, Chou et al. had done a meta-analysis identifying qualitative NMP22 which has a sensitivity of 69% and specificity of 77% and qualitative NMP22 has 83% in specificity and 70% in sensitivity (6). A meta-analysis by Wang et al. in 2017 showed a pooled sensitivity from of 56% and specificity of 88% for bladder cancer detection from 19 studies (7). However the sensitivity was low when tumour stage and grade were considered with sensitivity increasing steadily with stage of 13.68, 29.49, and 74.03% for Ta to T1 and >T2, respectively. It was also found to have a better diagnostic performance in the Asian population. NMP22 measures the cell turnover that occurs with surface shedding from bladder tumour. This process occurs in benign conditions such as inflammation, infection, bladders stones and haematuria thus resulting in false-positive results.

### Bladder Tumour Antigen (BTA) Assays

BTA tests detect human complement factor-H related protein in the urine which is produced by our bodies to protect cells from complement activation. It has an almost identical structure to the complement factor-H related protein produced by bladder cancer cells. There are two forms of BTA assays: (1) BTA Stat test: a “point-of-care (POC)” immunochromatographic assay which utilises five drops of urine to deliver a result within 5 min, and (2) BTA-TRAK test: standard quantitative ELISA measurement of the antigen. The FDA have approved them both for surveillance in BC in conjunction with cystoscopy only.

A meta-analysis conducted reviewing 13 studies identified specificity and sensitivity of BTA stat test to be 67 and 75%, respectively (8). Although BTA stat had shown higher sensitivity that urine cytology, the latter had better specificity. Chou et al. reviewed 22 studies identifying the sensitivity of BTA STAT was 64 % and specificity was 77%. For BTA-TRAK, four studies were evaluated and had similar results with a sensitivity of 65% and specificity was 74% (6). Similarly to other biomarkers, sensitivity had a positive correlation with increasing tumour grade of the BC.

Overall, the sensitivity and specificity of BTA Stat test ranges from 56 to 83% and 64 to 86% and with specificity up to 93% in individuals with benign conditions (9–12), and with BTA TRAK this ranges from 62 to 76% and 51 to 98% (13). Glas et al. carried out a meta-analysis and results of the bivariate analysis showed a sensitivity and specificity of cytology, BTA-Stat and BTA TRAK to be the following 55 and 94%; 70, 75, and 66%, and 65%, respectively (14). Given their lower specificities and similar issue of false positive results in benign conditions such as previous intravesical therapy, kidney stones, infection and presence of ureteric stents or nephrostomy tubes, theses tests are unable to replace cytology and can only be used concurrently as part of surveillance (9, 12, 15, 16).

### UroVysion

UroVysion is a molecular test using multicolour fluorescence *in situ* hybridisation (FISH) assay to detect aneuploidy of chromosomes 3, 7, and 17 and loss of the p16 gene at the 9p21 locus which are genetic abnormalities seen in BC. A sample must have a minimum of 25 cells to be analysed and a positive test is defined by one of the following: (1) Four or more morphologically abnormal cells have polysomy of two or more chromosomes (3, 7, or 17) (2) ≥10 cells with gain of a single chromosome (3) homozygous deletion of 9p21 in 12 cells (16).

Pooled results from a meta-analysis of 13 studies showed a specificity of 83% and sensitivity of 72% in comparison to urine cytology with 96 and 42%, respectively (17). UroVysion showed the sensitivity and specificity of 75.6 and 84.8%, respectively for high grade UCC, 40.8 and 87.8%, respectively for lowgrade UCC (18). Thus it faced a similar challenge to biomarkers in detection of low grade or low stage tumours (19). However the advantage of this assay is the absence of benign conditions such as cystitis, inflammation or haematuria affecting results.

In surveillance, Yoder et al. identified over a period of 29 months that 65% of the cases with positive UroVysion but no visible lesions developed recurrence on follow-up but this was not the case for Dimashkieh et al. as where 46% (158 of 343) of patients who had positive UroVysion tests did not develop UCC during up to the 3 year follow-up (18, 20). Therefore there is variability in its clinical utility. There is also evidence to show its potential in assessing response to intravesical BCG therapy for NMIBC (21).

### Immunocyt/Ucyt + Test

The Immunocyt/Ucyt+ test uses three fluorescent monoclonal antibodies (M344, LDQ10, and 19A211) which detect carcinoembryonic antigen and sulphated mucin glycoproteins on exfoliated urothelial cells in voided urine. Compoj et al. evaluated 7,244 cases and identified an overall sensitivity of 34.5% for cytology and 68.1% for uCyt+/ImmunoCyt and 97.9% for cytology, 72.3% for uCyt+/ImmunoCyt (22). There was a positive correlation with higher grade and specificity along with sensitivity as observed with other biomarkers. A meta-analysis identified a sensitivity of ImmunoCyt to be 75% and specificity was 78% and in comparison to NMP22, BTA and FISH, it had the highest pooled sensitivity (6). A split study comparing UroVysion, ImmunoCyt and cytology supported this with Immunocyst being more sensitive in detecting low grade tumours (23). Further meta-analysis also supported previous evidence to support the use of Immunocyt in combination with cytology in surveillance to reduce the frequency of follow up required in low risk cancers (24).

However, this test involves advanced technical expertise as a minimum of 500 cells need to be analysed for fluorescence to provide accurate results and thus there is interobserver variability and need for high cellularity specimens. It can be affected by benign conditions such as haematuria albeit not as easily as other biomarkers above (25).

## Promising Protein Biomarkers

Table 1 summarises potential protein biomarkers which can be used in BC surveillance highlighting meta-analysis and most relevant study in the table.

**Table 1:** Additional protein biomarkers.

**Biomarker**	**Description**	**Method**	**N**	**C**	**SS**	**SP**	**Comment**	**Ref**
**UBC**	Detects the presence of fragments of cytokeratin 8 and 18 in urine	ELISA or immunoradiometric assay	753	1072	64.4	80.3	UBC values higher in high-grade tumours and able to distinguish from low-grade. Higher specificity in combination with cytology or survivin assay	(26–29)
**CYFRA21-1**	Quantifies soluble fragments of cytokeratin 19	ELISA	1262	1233	82.0	80.0	Significantly higher levels in patients with metastatic disease vs. locally invasive unable to differentiate between histological grades	(30, 31)
**BLCA-4**	Measures protein components of the nuclear matrix which are present in the urothelium of BC patients	ELISA	1119 total participants		93.0	97	Meta-analysis mainly retrospective studies showing potential to detect early tumours. No positive correlation between tumour stage and levels measured.	(32, 33)
**CellDetect**	Composed of a unique plant extract which interacts with malignant cells due to their increased metabolic activity	Immunostain	84	110	84.0	70.0	Two studies have shown higher sensitivity in low grade tumours in comparison to urine cytology (82% vs 59%) and similar specificity (86 vs 94%) It was also found not be affected by haematuria.	(34, 35)
**Hyaluronic acid**	HA is a glycosoaminoglycan and HAse is endoglucosidase involved in tumour metastases and breakdown of HA into fragments for angiogenesis	ELISA and RT-qPCR	918 participants		90.8	82.5	In comparison to BTA stat, UBC and cytology in two studies shown superior SS and SP. SS and SP not affected by tumour grade but levels are not indicative of tumour grade. More studies required to evaluate this promising marker.	(36–38)
**sFas**	Anti-apoptotic protein released by BC cells to protect from anti-tumour activity	ELISA	128	88	51.2	85.9	Lower sensitivity. Higher levels associated with higher risk of recurrence.	(39–41)
**Survivin**	Overexpression in BC as a protein which inhibits apoptosis pathways	Bio-dot test	50	44	82	90	Limited data available in follow up setting or in comparison to other biomarkers	(42)
**MCM5 - ADXBladder**	Detects MCM5 shed by replicating BC cells	ELISA	503 patients		51.9	66.4	Findings in prospective study in comparison to UC with SS 16.9% and SP 98%. Low sensitivity for low grade tumours. 99% NPV for high risk NMIBC	(43)
**URO17**	Detects oncoprotein Keratin 17 involved in the replication of cancer cells	Immunocyto-chemistry	81	98	97	AUC: 90	Consistently high sensitivity and specificity from 3 independent studies. Good potential as simple incorporation to existing equipment	(44–46)

*N, tumour samples; C, control; SS, sensitivity; SP, specificity; UBC, Urinary bladder cancer; BLCA-4, Human Bladder Cancer-associated Nuclear Matrix Protein4; HA, hyaluronic acid; HAse, hyaluronidase; sFas, soluble Fas; RT-qPCR, Real-Time Quantitative Reverse Transcription; BC, bladder cancer; UC, Urine cytology; NPV, negative predictive value; NMIBC, Non-muscular invasive bladder cancer; AUC, Area under curve*.

The UK National Health Service (NHS) approved the usage of ADxBladder in BC detection. Three prospective studies have been done with only one in the surveillance setting (43). They reported an overall sensitivity ranging between 45–73%, specificity between 70–73%, and NPV between 74–100% and were superior to cytology (47). Given the turnaround time of 2h, it being relatively unaffected by benign conditions such as inflammation or haematuria and consisting of a single biomarker identifiable with ELISA which is readily available in labs and costing only £0.37 per person, this made ADxBladder a viable option (48). However, in comparison to other biomarkers identified, it has a low sensitivity and specificity as displayed in Table 1 and poor performance in detection of low grade tumours.

Of the new protein biomarkers that were introduced in recent years, URO17 test utilising Keratin 17 (K17) has shown especially promising results. Babu et al. had identified high sensitivity and specificity of URO17 of 100% using urine samples in a retrospective study (44). Interestingly, URO17 is able to detect both low and high grade cancers in patients presenting with haematuria, a previously excluded cohort, thus proving its benefit of use in a surveillance setting as well (45). There is a specificity of 96 and 92.7% in recurrent and newly diagnosed patients, respectively (44, 45). Given these outcomes and its easy adaptation to current equipment used, a larger prospective study in a surveillance setting would be beneficial in developing this into a promising protein biomarker test for non-invasive surveillance of NMIBC (45, 46).

## Gene-Related Biomarkers

Genetic alterations has been explored as another avenue for detecting bladder cancer in surveillance. To discuss this further we will divide these into the following groups: DNA methylation markers, histone tail modifications, miRNA biomarkers, microsatellite analysis and multi-gene panels.

Table 2 summarises detection of genetic alterations to utilise as biomarkers listing the most relevant study accounting for those with the largest patient groups and most representative of the target group.

**Table 2:** Gene-related biomarkers.

	**Biomarker**	**Description**	**M**	**N**	**C**	**SS**	**SP**	**Comment**	**Ref**
**4.1**	*FGFR3, TERT* and *OTX1*	Combination of DNA methylation levels and DNA mutation analysis	SNaPshot®	977 pts 2496 sp		LG: 57% HG: 72%	LG: 59% HG 59%	Large prospective study identified lower sensitivity in follow up HG and LG patients in comparison to primary LG and HG. Increased sensitivity with HG tumours.	(49)
	HS3ST2, SEPTIN9, and SLIT2/FGFR3	Detects 4 hotspot mutations in FGFR3 in combination with DNA methylation levels	qMS-PCR	157		63	58	Combination with methylation shows sensitivity of 94% in low grade and 100% in high grade to 100% in recurrence	(50)
	SOX-1, IRAK3, and Li-MET [	Detecting changes in DNA methylation in BC cells shed in urine		90		86	89	Tumour recurrence predicted in 80% of patients, superior to cytology (35%) and cystoscopy (15%)	(51)
	APC _ a, TERT _ a, TERT _ b, and EDNRB		MS-MLPA	49	60	72	55	All HG recurrent tumours including CiS were detected but pTa G1-2 were missed	(52)
**4.2**	Histone tail modifications (HTF) H3K9 and H3K27	HTFs help regulate cell processes which are fundamental to DNA repair. Low levels have been associated with BC	Immuno-histochemical analysis	113	61	N/A		H3K9 and H3K27 levels correlate with invasiveness of BC along with grade and pT stage of NMIBC, Expression of H3K27me3 predictor for cancer-specific survival post-cystectomy.	(53)
**4.3**	Has-let-7c, miR-135a, miR-135b, miR-148a, miR-204, miR-345	Analysis of 364 different miRs was analysed in 16 urine samples to identify a 6 gene signature	miRNA assay	130‘	112	AUC 88.3%		AUC of 88.0%, 92.9% and 91.0%, for LGNMIBC, HGNIMBC and MIBC respectively. Validation sets were done. Newly diagnosed patients only, limiting clinical applicability in surveillance.	(54)
	6 step: **miR-187, miR-18a, miR-25**, *miR142-3p, miR-140-5p, miR-204* 2 step: miR-92a, miR-125b	In bold, overexpressed in BC and in italics, they were under expressed.	miRNA assay	27	10	85	87	2 step model predicts progression and cancer specific survival in NMIBC patients. However, only a small case control study	(55)
						85	74		
	miR16, miR200c, miR205, miR21, miR221 and miR34a	Panel of 12 miRNAs profiled and identified set of 6. Validation study included.	RTq-PCR	81	21	88	48	AUC 0.85 in predicting recurrence in surveillance setting. Better performance in larger tumours and higher T-stage	(56)
	miR-21, miR-15a, miR-200c miR-93, miR-191, and miR-940, Has-let-7b	Seven miRNA identified to have significantly higher levels in the cancer group	RTq-PCR	85	45	88	78	No validation study. Highest sensitivity in T1G3 and >T2 patients.	(57)
	25 target diagnostic miRNA signature	A panel of 46 miRNAs monitored in an independent cohort of 121 subjects identifying 25-target panel	RTq-PCR	60	61	87	100	No validation cohort. Further prospective studies required.	(58)
**4.4**	Xpert Bladder	Detects expression levels of 5 mRNA expression of genes (CRH, IGF2, UPK1B, ANXA10, and ABL1)	LDA	239	-	74	80	Prospective study. Higher SN and NPV compared with cytology and UroVysion with NPV of 98% in HG disease	(59)
	AssureMDX	Mutation analysis in FGFR3, TERT, and HRAS genes and methylation analysis in OTX1, ONECUT2, and TWIST1 genes	SNaPshot® + MS-PCR	74	80	97	83	Follow up validation study showed SS of 93 and SP of 86. AUC higher in >Ta compared to Ta and om HG tumours	(60, 61)
	CxBladder	IGFBP5, HOXA13, MDK, and CDK1 are associated with carcinogenesis and the 5th biomarker (CXCR2) is a marker of inflammation reducing false positive results	Rt-qPCR	66	417	82	85	97% SS in HG tumours and 69% in Ta. Greater SS in comparison to urine cytology and NMP-22	(62)
	EpiCheck	Blinded, single-arm, prospective multicenter study using Epicheck (15 proprietary DNA methylated genes) in NMIBC surveillance	Rt-qPCR. EpiScore	353	-	68	88	SS 100%, 40%, 89% in Cis, LG and HG respectively.	(63)
	Uromonitor	FGFR3 hotspot and TERT promotermutations	Rt-qPCR	122	-	73	73	Addition of KRAS mutation to UroMonitor V2 kit, increased SS to 100% and specificity to 83.3%	(64)
	UroSEEK	Mutations in 11 genes or presence of abnormal number of chromosomes	NGS	496	-	74	72	UroSEEK positivity preceded tumour recurrence diagnosis by 4 months in average. However, this is a retrospective study and prospective studies needed.	(65)

*M, Method; N, number of malignant samples or cancer patients; C, control; SS, sensitivity; SP, specificity; Ref, reference; LG, low grade; HG, high grade; Cis, Carcinoma in situ; qMS-PCR, quantitative methylation specific-polymerase chain reaction; Rtq-PCR, Quantitative reverse transcription PCR; AUC, area under curve; NGS, next-generation sequencing assay; LDA, linear discriminate analysis*.

### DNA Methylation Markers

Epigenetic alterations are part of the carcinogenesis. DNA hypermethylation of the CpG islands play a role in the promoter regions of tumour-suppressor genes. This mediates silencing of the affiliated gene which is a known phenomenon in BC. The hyper or hypomethylation of these genes can be detected in tumour cells that are shed into urine and aid diagnosis of BC. This has been reviewed in both primary and recurrent tumours. Bosschieter et al. evaluated 42 studies and identified 8 with high sensitivity and specificity with varying methodologies and heterogenous patient groups (66). Studies with promising results, had no independent validation data and Costa et al. with 94% specificity and 90% sensitivity did not report on tumour grade or stage (67). This is relevant as similarly to other reported biomarkers, results will vary with disease spectrum. As shown in Table 2 section DNA Methylation Markers studies in recurrence are listed but these are mainly small retrospective case-control studies (50). Beuker at al, as shown in Table 2, used a combination of this technique along with DNA mutation analysis with FGFR3 and TERT mutation analysis to improve detection rates showed similar results as increased sensitivity in high grade tumour cells likely due to increased shedding of BC cells (49). There is an insufficient amount of data due to variability in methodology, patient groups and gene panel selection. Studies have used it in combination with DNA mutation analysis.

### Histone Tail Modifications

Other than epigenetic changes described above, another manifestation of this is histone lysine methylation (HxKy). Histone modifications help regulate numerous cell mechanisms such as chromosome condensation, DNA repair and transcription. The site and degree of histone methylation determines the transcriptional activity. H3K9, as mentioned in Table 2 section Histone Tail Modifications are associated with repressed transcription. Other potential histone modications identified are H3K4 and H3K20 methylation which were decreased in BC compared to normal patients and global H4K20me3 levels were predictive for bladder cancer-specific survival (68).

### miRNA Biomarkers

Micro RNAs (miRNA) are short noncoding RNAs that regulate process post-transcriptionally and dysregulation leads to carcinogenesis. The aberrant expression of miRNA has led to its potential use as a biomarker. It can present in bodily fluids as they are protected by RNAse degradation because they are excreted as membrane-protected free circulating miRNAs or in extracellular vesicles (EVs) such as exosomes (69). Initially identification was done using qtPCR but now rapid profiling is done using microassays and miRNA sequencing (56).

Studies listed in Table 2 section miRNA Biomarkers are those with sensitivities of more than 80%. Multiple miRNA diagnostic assays had better sensitivity than single miRNA assays. Chen et al. carried out a meta-analysis 30 studies with 1019 BC patients and 690 controls identifying a pool sensitivity and specificity of 80 and 74%, respectively (70). The AUC for NMIBC was 0.84 and 0.76 for MIBC suggesting higher diagnostic ability in NMIBC patients. Another meta-analysis by Shi et al. evaluated 1,556 cases and 1,347 controls from 31 studies with a pooled specificity and sensitivity of 72 and 76%, respectively (71).

Most studies compared a heterogenous group of BC patients with controls. Study which explored the recurrence setting in both NMIBC and MIBC using miR-145 and miR-200a. This identified a sensitivity of 78% and specificity of 61% in NMIBC patients where lower levels of miR-145 associated with higher grade and lower levels of miR-200a independently predicted recurrence (72). Prospective trials in BC surveillance are required to validate the clinical applicability of this biomarker.

### Multi-Gene Panels

Several assays detecting mRNA biomarkers have been conducted. Table 2 section Multi-Gene Panels summarises them. CxBladder has been extensively studied and has variations which include: (1) Cxbladder® *Detect* to detect bladder cancer in hematuria patients with a sensitivity of 82% and specificity of 85% (62). (2) Cxbladder® *Triage* which is used in hematuria patients to rule out BC with a sensitivity of 95% and negative predictive value of 97% (73). (3) Cxbladder® *Monitor* as a complement to surveillance. It has been compared with urine cytology, NMP22 BladderChek and NMP22 ELISA with a superior SS and SP of 91/96 vs. 22/87%, 11/87 and 26/86 % (74). Koya et al. implemented CxBladder Monitor (CxBM) into local guidelines whereby low risk patients had alternate annual CxBM and cystoscopy therearfter (75). They found that 77.8% of patients were safely managed by only one cystoscopy every 2 years, reducing the total number of annual cystoscopies by 39%. This was reflected in a real world data analysis identifying this advantage of CxBM in clinical practice to have driven its increased utility (76).

### Other Possible Gene-Related Biomarkers

Microsatellite analysis (MSA) through PCR targets highly pleomorphic short tandem repeats (STR) which occur in cancer cells with loss of heterozygosity (LOH) causing microsatellite instability. This occurs because of epigenetic silencing or inactivation of the mismatch repair gene which play an integral part in the proliferation of cancer cells. The most common LOH is in chromosome nine but this is also seen to occur in chromosome 4, 8, 11, and 17p (77–80). In comparison to urine cytology, sensitivity was 97 vs. 79% with 95–100% in low grade tumours in a small study of 34 cancer patients with 21 cancer-free subjects (81). A prospective study of 228 patients undergoing BC surveillance had a specificity and sensitivity of 58 and 74%, respectively (82). A further prospective study of 91 patients evaluating MSA in combination with cytology had a sensitivity of 72% in G1–2 and 96% in G3. They found using LOH analysis to improve specificity and all recurrence cases were identified (83). Overall, this test has good sensitivity but difficult to incorporate into present laboratories due to its complexity. Larger prospective studies which include a validation cohort to assess feasibility is required.

## Extracellular Vesicles and Exosomes

Exosomes are vesicles secreted by cells which mediate extracellular communication by transmitting proteins, lipids, miRNA, mRNA and long non-coding RNAs (lncRNA). Extracellular vesicles in BC cells carry a vast number of proteins which is utilised in angiogenesis and cell migration thus aiding further tumour progression which can be utilised as markers but possible novel therapeutic targets (84). Different analytical methods have identified various proteins and miRNAs in this rich extracellular environment. A small study isolated EV using microchip ELISA and found highly elevated EV levels in BC patients compared to controls (85). Profiling of proteomes in exosomes identified a correlation with tumour grade and the ability to predict recurrences with 1-antitrypsin and histone H2B1 exosome proteins (86). In another study, urinary EVs were isolated and deregulated miRNAs were identified as potential biomarkers, in particular, miR-375 for high-grade bladder cancer while miR-146a for low-grade patients (87).

EVs are promising as a source of biomarkers given the diverse cargoes EVs carry. However, there are several limitations such as isolation techniques and testing not being standardised and therefore it is difficult to compare results between groups given lack of reproducibility. Studies conducted are heterogenous and small without any validation sets (88). Optimisation of testing could lead to better EV studies allowing for real EV biomarker development in a clinical setting and could further inform us further on tumour biology.

## Conclusion

This paper has highlighted the various biomarkers in urothelial cancer and their significance in early diagnosis of bladder cancer. Whilst it's important to have biomarkers in NMIBC, differences in sensitivity and specificity limits their use in the community. These biomarkers have a significant role in future diagnosis of bladder cancer, and future studies will guide clinicians in using the most appropriate marker for screening. The advancement in analytical methods in BC has driven the research towards non-invasive liquid-based biomarkers in adjunct to urine cytology. This paper provides evidence that a second modality of screening tool may be beneficial to use in the diagnostic algorithm for bladder cancer. Studies identifying its feasibility in a clinical enviroment is important as they have limitations when used in isolation. Given this is a narrative review, further evaluation of these promising markers is required in more depth in terms of a meta-analysis along with the development of prospective studies in a surveillance setting. Meta-analyses on the newer markers have not been conducted as there is variability in patient cohorts utilised and more studies need to be conducted to obtain sufficient data. In addition to this, majority of studies are in a restrospective setting and prospective studies need to be developed to be able to further evaluate their clinical feasibility.

The ongoing pandemic has further accentuated the increasing need and relevance for biomarkers to cope with delay in cystoscopies in both diagnosis and surveillance. The use of more sensitive methods to detect true tumour recurrences could also play a role in assessment of the diagnostic accuracy of these markers, potentially reducing the number of cystoscopies for test-negative cases and introducing methods like blue light cystoscopy for test-positive cases. Further large, prospective clinical trials incorporating these biomarkers and usage of newer analytical methods in screening for high-risk patients and also in disease recurrence in will allow for its use in a clinical setting.

## Author Contributions

SS collated and summarised studies to write up majority of this review. KN and AN were involved in editing. AS and NV contributed to the introduction and conclusion. All authors contributed to the article and approved the submitted version.

## Conflict of Interest

The authors declare that the research was conducted in the absence of any commercial or financial relationships that could be construed as a potential conflict of interest.

## Publisher's Note

All claims expressed in this article are solely those of the authors and do not necessarily represent those of their affiliated organizations, or those of the publisher, the editors and the reviewers. Any product that may be evaluated in this article, or claim that may be made by its manufacturer, is not guaranteed or endorsed by the publisher.
